# Supplementary material for: The Construction and Exploration of a Comprehensive MicroRNA Centered Regulatory Network in Foxtail Millet (Setaria italica L.)
Source: Front Plant Sci. 2022 May 6;13:848474. doi: 10.3389/fpls.2022.848474 (PMC9121102; doi:10.3389/fpls.2022.848474)
Supplement: Supplementary file 1 [file Data_Sheet_1.zip › Sit-miR827_Seita.9G188600.1_6083_TPlot.pdf]

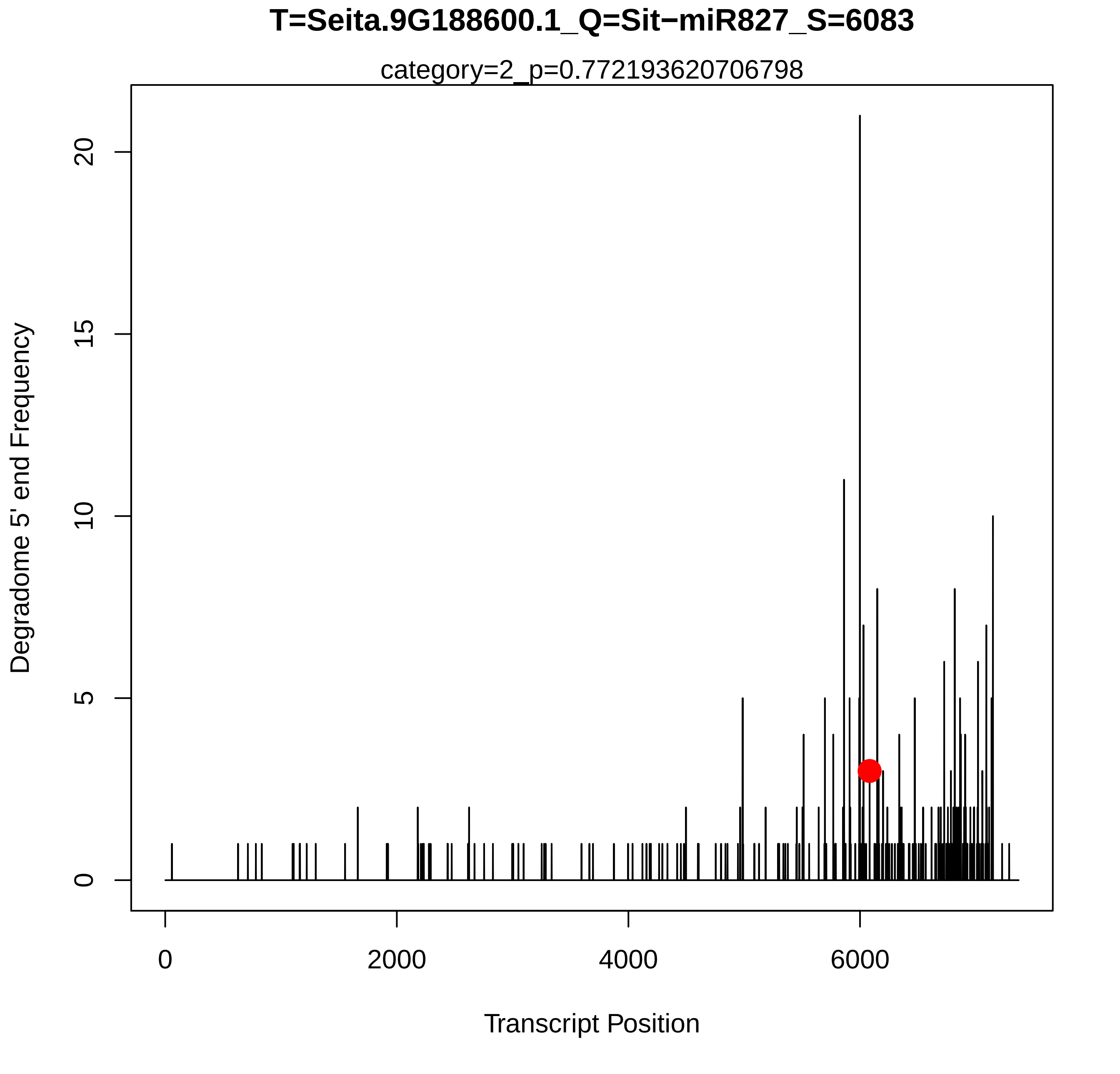
T=Seita.9G188600.1_Q=Sit−miR827_S=6083
category=2_p=0.772193620706798
Degradome 5' end Frequency
0
5
10
15
20
0
2000
4000
6000
Transcript Position
